# Characteristics and Ablation Outcomes of Atrial Tachycardia in Patients with Prior Cardiac Surgery vs. Spontaneous Scars: Where Are the Differences?

**DOI:** 10.3390/jcm11185407

**Published:** 2022-09-15

**Authors:** Junqi Wang, Sainan Li, Ming Liang, Mingyu Sun, Zhiqing Jin, Jian Ding, Yaling Han, Zulu Wang

**Affiliations:** Department of Cardiology, General Hospital of Northern Theater Command, Shenyang 110016, China

**Keywords:** atrial tachycardia, cardiac surgery, spontaneous scar, catheter ablation

## Abstract

(1) Background: Atrial scars play an important role in atrial tachycardia (AT). They can not only be found in patients with prior cardiac surgery (PCS) but also in patients without PCS or significant structural heart disease, in which case the scar is called a spontaneous scar (SS). This study aims to compare the characteristics, mechanisms and ablation outcomes of AT in patients with PCS and SS. (2) Methods: We retrospectively reviewed electrophysiological and ablative characteristics of ATs in 46 patients with PCS and 18 patients with SS. (3) Results: There were averages of 1.52 and 2.33 ATs per patient in the PCS group and SS group, respectively (*p* < 0.01). Cavo-tricuspid isthmus dependent atrial flutter (CTI-AFL) was presented in most patients in both groups (93.50% vs. 77.80%, *p* = 0.17), whereas the SS group had a higher occurrence of scar-mediated reentrant AT (SMAT) and focal AT (FAT) compared with the PCS group (88.90% vs. 39.10%, *p* < 0.01; 22.2% vs. 2.2%, *p* < 0.05). There were no significant differences in acute success rate between the two groups, whereas patients with SS had lower long-term success rate (87.0% vs. 61.1%, *p* < 0.05) and higher occurrence of sinus node dysfunction (SND) (4.3% vs. 22.2%, *p* < 0.05). (4) Conclusions: CTI-AFL is common in both patients with PCS and SS, and routine CTI ablation is recommended. Compared with patients with PCS, patients with SS have more ATs, especially with higher occurrence of SMAT and FAT, and had a lower long-term success rate and higher incidence of SND.

## 1. Introduction

With the advances in surgical procedures, the life spans of patients with congenital heart disease or other severe structural heart diseases have been improved. However, some long-term complications, such as atrial tachycardia (AT), ensue. Surgical scars, surgical patches and atrium fibrosis in patients with prior cardiac surgery (PCS) provide the acquired atrial pathological substrate and facilitate the occurrence of AT [1,2]. However, some patients without PCS or structural heart disease can also have atrial scars, which are called spontaneous scars (SS) [3,4]. These scars, usually diffused and scattered, also play an important role in the occurrence of AT [3,4,5]. Some research demonstrated that cavotricuspid isthmus dependent atrial flutter (AFL) and scar-mediated reentrant atrial tachycardia (SMAT) were important mechanisms for AT in patients with PCS [6,7,8,9], whereas similar results were also found in SS patients [10]. Therefore, this research aims to compare the characteristics and mechanisms of ATs in patients with PCS and SS, as well as their long-term outcomes after catheter ablation.

## 2. Methods

### 2.1. Population

Sixty-four patients with right-sided AT were retrospectively reviewed, all of whom had undergone AT ablation at General Hospital of Northern Theater Command from September 2013 to April 2019. Those with left-sided AT were excluded. Among these 64 patients, 46 patients with a history of cardiac surgery were classified as the PCS group, and 18 patients with a right atrial scar but without a history of invasive cardiac surgery or structural abnormalities were classified as the SS group. Demographic characteristics, past histories and electrocardiograms were recorded. All available surgical recordings, echocardiograms and chest X-rays were collected. The study was conducted in accordance with the Declaration of Helsinki, and approved by the Institutional Ethics Committee of General Hospital of Northern Theater Command [protocol code: Y(2022)075].

### 2.2. Electrophysiologic Study and Electroanatomic Mapping

All patients underwent electrophysiological study after written informed consent. All antiarrhythmic drugs except for amiodarone were discontinued for at least 5 half-lives prior to the procedure. Electrophysiological study and ablation were performed under conscious sedation with fentanyl. A decapolar catheter was advanced to the coronary sinus though the femoral or jugular vein and a quadrupolar catheter was positioned in the His bundle area via femoral access in all patients. If patients presented sinus rhythm at the beginning of procedure, arrhythmia was induced by burst atrial pacing or programmed atrial stimulation, and isoproterenol was used when necessary. An ablation catheter (Navistar ThermoCool, Biosense-Webster, Diamond Bar, CA, USA), PentaRay multipolar catheter (Biosense-Webster, CA, USA) or a 64-electrode basket-catheter (Orion, Boston Scientific, Cambridge, MA, USA) was used for atrial mapping. Electroanatomic mapping of the right atrium was performed under tachycardia using the Carto Mapping System (Biosense Webster Inc., Diamond Bar, CA, USA) or the Rhythmia HDx Mapping System (Boston Scientific, Cambridge, MA, USA). The area with bipolar potential ≤ 0.05 mV was viewed as an atrial scar and 0.05–0.5 mV was defined as the low-voltage zone (LVZ). A macro-reentrant AT was considered when activation mapping showed a continuous atrial activation sequence with the “early meets late” pattern and the activation time > 90% of the tachycardia cycle length. AFL was diagnosed when the macro-reentrant circuit rotates around the tricuspid annulus (TA) in a clockwise or counterclockwise direction, and entrainment at the lateral side of TA and coronary sinus ostium showed post-pacing interval (PPI)–total cycle length (TCL) < 30 ms. SMAT was defined as macro-reentrant AT circuiting around the atrial scar. Entrainment was performed in the regions recording low-amplitude high-frequency fragmented potential, and the entrainment point in the critical isthmus was suggested when concealed fusion was seen and PPI–TCL < 30 ms. A focal AT (FAT) was diagnosed by a radial spread of atrial activation from a distinct focus on the activation mapping, with markedly short atrial activation duration compared with the cycle length (CL).

### 2.3. Ablation

**Figure 1:**
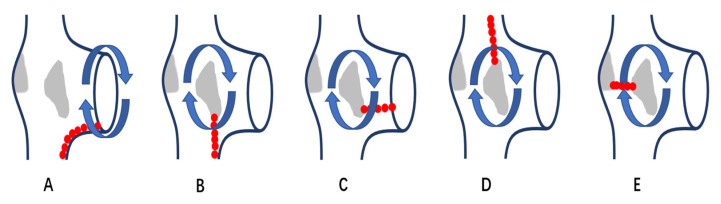
Schematic diagram of different ablation position of reentrant AT in patients with right atrial scars. (**A**): a diagram of AFL, which is a reentrant tachycardia rotating around TA in clockwise or counterclockwise direction; linear ablation is performed from TA to IVC. (**B**–**E**): diagrams of SMAT, which are reentrant tachycardias circuiting around atrial scars; linear ablation is performed from scar to IVC in (**B**), scar to TA in (**C**), scar to SVC in (**D**) and between scars in (**E**). AFL: cavo-tricuspid isthmus dependent atrial flutter; SMAT: scar-mediated atrial tachycardia; IVC: inferior vena cava; TA: tricuspid annulus; SVC: superior vena cava.

Radiofrequency (RF) was applied with an irrigated ablation catheter at a temperature of 43–45 °C, power of 30–40 W and saline flow of 17–20 mL/min. For macro-reentrant AT, after the tachycardia circuit was identified by carefully mapping, RF ablation was performed at the narrowest identifiable critical isthmus of the circuit or between two appropriate barriers. Linear ablation from TA to inferior vena cava (IVC) was performed in patients with AFL. For SMAT, ablation was performed between scar and anatomical obstacle, such as TA, IVC and the superior vena cava (SVC), or between two scars (Figure 1). For FAT, ablation was targeted at the earliest activated area. Ablation was considered acute success when no AT could be reinduced under atrial programmed stimulation or burst atrial pacing, even with isoproterenol administration when necessary, and bidirectional conduction block of the ablation line was confirmed by pacing mapping or activation mapping.

### 2.4. Follow Up

Routine follow-ups were performed at 1, 6 and 12 months after procedure at an outpatient clinic and once a year thereafter. Electrocardiogram or 24-h Holter was performed at the outpatient clinic visit and at any time patients experienced symptoms. However, for patients living far away from this city and unable to receive clinic visits, an electrocardiogram or 24-h Holter were performed in local institution and examination results were transmitted by telephone or social software WeChat. Critical events such as recurrence or sinus node dysfunction (SND), ICD implantation and redo ablation were recorded.

### 2.5. Statistical Analysis

Continuous variables are expressed as mean ± SD and compared using Student’s t-test (parametric distribution), or median and quartiles (M1, M3) and compared using the Mann–Whitney U test (nonparametric distribution). Categorical variables are summarized by percentages and frequencies and are compared using the Chi-square test or Fisher exact test except for ordinal categorical variables, which are compared by the Mann–Whitney U test. *p* value < 0.05 was considered statistically significant. Analyses were performed with SPSS software version 26.0 (SPSS Inc., Chicago, IL, USA) for Windows.

## 3. Results

### 3.1. Patients’ Characteristics

Among 46 patients with PCS (median surgery age 22.5 years old), 35 patients had repaired congenital heart diseases, most of whom had congenital heart diseases of moderate (20 [57.1%]) or severe (3 [8.5%]) complexity according to the ACHD guideline [11]. Eleven patients underwent cardiac surgery for non-congenital heart diseases, 7 with valvular heart disease and 4 with benign cardiac tumor. The types of initial structural heart disease in patients with PCS are listed in Table 1.

In 18 patients with SS, no patients had significant structural abnormality. The age of ablation in patients with SS were older than those with PCS (median age: 46.00 vs. 56.50, *p* < 0.01). There were no significant differences in gender, hypertension, diabetes mellitus, sustained AT and prior catheter ablation between two groups (*p* > 0.05). The diameter of the right ventricle and the longitudinal diameter of right atrium were longer in the PCS group (*p* < 0.01). The baseline characteristics of patients with PCS and SS are shown in Table 2.

### 3.2. Characteristics of Atrial Tachycardias and Ablation

**Figure 2:**
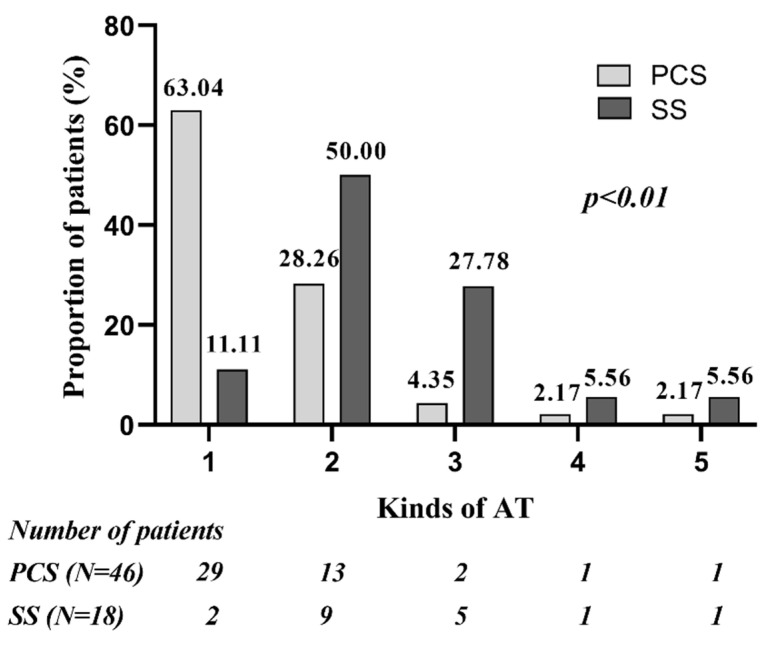
Quantitative analysis of ATs in patients of PCS group and SS group. Compared with PCS group, larger proportion of patients in SS group had multiple ATs. PCS: prior cardiac surgery; SS: spontaneous scar.

**Figure 3:**
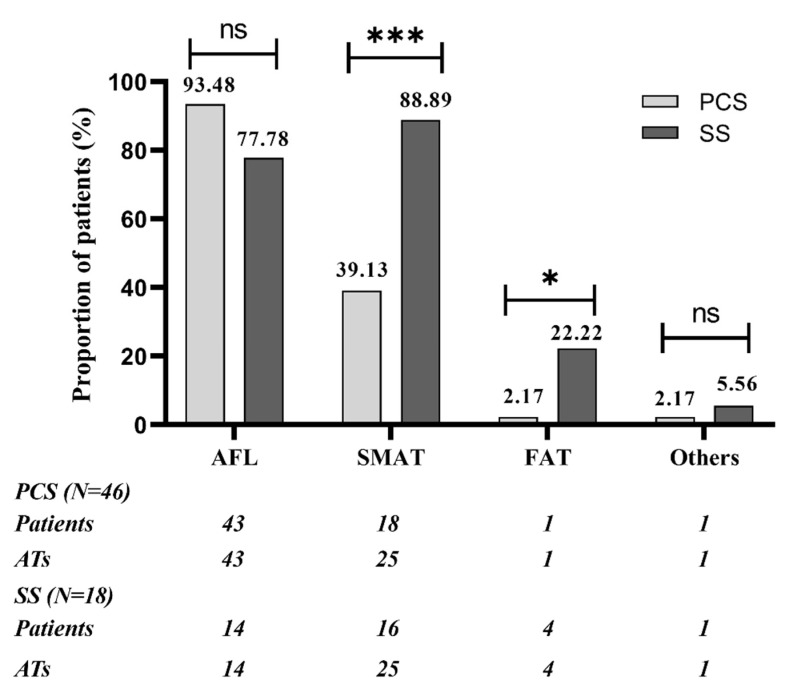
Qualitative analysis of ATs in patients of PCS group and SS group. SMAT was the most common AT in SS group, with significantly higher occurrence than in PCS group; AFL was the most common AT in PCS group, whereas there were no significant differences between 2 groups. FAT was rare, but the SS group compared with PCS group had higher occurrence of FAT. SVC/IVC-reentrant tachycardias were classified as “others”; 1 patient in PCS group had SVC-reentrant tachycardia and 1 patient in SS group had IVC-reentrant tachycardia. AFL: cavo-tricuspid isthmus dependent atrial flutter; SMAT: scar-mediated reentrant atrial tachycardia. * *p* < 0.05. *** *p* < 0.001. ns: not significant.

There was an average of 1.52 (70/46) and 2.33 (42/18) kinds of spontaneous or induced AT in patients of PCS and SS group, separately (*p* < 0.01). In the PCS group, most patients (63.0%) had only 1 kind of AT; AFL was the most frequent AT, found in 93.5% of patients, followed by SMAT, found in 39.1% of patients. In the SS group, most patients had multiple ATs, and both SMAT and AFL was presented in a high percentage of patients (88.9% vs. 77.8%, *p* > 0.05). The SS group compared with the PCS group had higher occurrence of SMAT and FAT (88.9% vs. 39.1%, *p* < 0.01; 22.2% vs. 2.2%, *p* < 0.05). FAT was found in 1 patient with PCS, located in the coronary sinus ostium, whereas in 4 patients with SS, located in the superior margin of the scar root of the right atrial appendage, nine o’clock of TA and SVC, separately. The quantitative and qualitative analysis of ATs in the two groups are presented in Figure 2 and Figure 3.

In the PCS group, 29 patients had only 1 kind of AT, including 27 patients with only AFL and 2 patients with only SMAT. In 27 patients had only AFL, all of them had scars in the right atrial free wall except for 2 patients who showed no apparent surgery-related scars, and septal scars were combined in 3 patients. CTI ablation from TA to IVC was performed in the 27 patients except for 1 patient with dextrocardia, in whom a large atrial scar was seen in the lateral wall of RA (in the left side) and ablation from a scar to the TA (in the left side) eliminated the tachycardia. The other 2 patients with only 1 kind of AT had reentrant tachycardia around lateral wall and ablation from a scar to IVC eliminated the tachycardia. Multiple ATs were presented in 17 patients in the PCS group. AFL combined with CSO-originated FAT was diagnosed in 1 patient and ablation in CSO successfully eliminated the tachycardia. And 1 patient with prior CTI ablation had 5 kinds of SMAT. In this patient, extensive scars were found in the lateral wall and anterior wall, and after extensive ablation, 1 kind of AT remained. Fifteen patients had both AFL and SMATs, of whom all had atrial scars in the free wall and 7 had dual-loop reentrant tachycardia. In these 7 patients, SMAT converted to AFL during ablation from a scar to IVC in 6 patients, and AFL converted to SMAT during CTI ablation in 1 patient. One patient had SVC reentrant tachycardia besides AFL and SMAT, which was eliminated by SVC isolation.

In the SS group, SMAT was found in 16 patients, including 13 combined with AFL. In these 16 patients, atrial scars were found in only lateral wall in 6 patients, in only the posterior wall in 6 patients, and in multiple atrial walls in 4 patients, including both septal and posterior walls in 2, septal and anterior walls in 1 and lateral and anterior walls in 1 patient. Linear ablation was performed from scar to IVC in 12 patients, scar to TA in 7, scar to SVC in 2, and between scars or within scar in 2. Three patients were combined with FAT, and the focal origins were in the SVC, 9 o’clock of TA, and root of the right atrial appendage (RAA), separately. In 2 patients without SMAT, 1 patient with lateral and anterior scars had FAT originating from the superior margin of the scar, and the other patient had both AFL and IVC reentrant tachycardia, in whom CTI ablation successfully eliminated the 2 kinds of AT, simultaneously.

### 3.3. Outcomes of Ablation and Long-Term Follow Up

**Figure 4:**
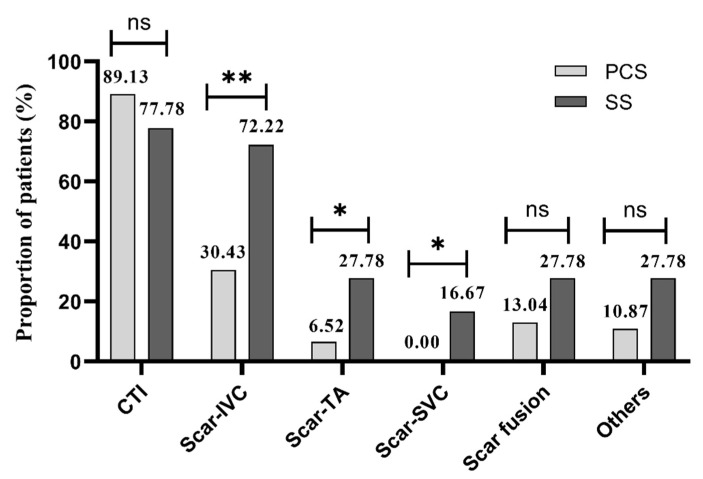
Ablation positions of 2 groups. PCS: prior cardiac surgery; SS: spontaneous scar; CTI: cavo-tricuspid isthmus; IVC: inferior vane cava; TA: tricuspid annulus; SVC: superior vane cava. * *p* < 0.05. ** *p* < 0.01. ns: not significant.

There was an average of 1.59 ablation positions in the PCS group and 2.89 in the SS group during the procedure (*p* < 0.01). CTI was the most common ablation position in both groups (89.1% vs. 77.8%, *p* > 0.05), but scar-related ablation was more frequently performed in patients with SS (from scar to IVC: 30.4% vs. 72.2%, *p* < 0.01; from scar to TA: 6.52% vs. 27.78%, *p* < 0.05; from scar to SVC: 0.0% vs. 16.67%, *p* < 0.05; Figure 4).

Ablation failed in 3 patients with PCS, 2 of whom had a large right atrium and multiple ATs (1 patient had a right atrial diameter of 60 mm and 3 kinds of Ats; the other had a right atrial diameter of 100 mm and 5 kinds of ATs) and 1 had ablation difficulty for abnormal cardiac structure. In the SS group, ablation failed in 3 patients, including 2 patients with ablation abandon for high risk of bradycardia and 1 with multiple atrial arrythmias. The acute success rates were 93.5% (43/46) and 83.3% (15/18) in the PCS and SS groups, respectively (*p* = 0.44).

During the long-term follow-up, 39 (84.8%) patients with PCS and 10 (55.6%) patients with SS were free from atrial arrhythmia (*p* < 0.05, Table 3). One patient with recurrence in the PCS group underwent surgical Maze procedure and 1 patient with recurrence in SS group underwent redo radiofrequency ablation, all of whom had no further recurrence at the end of the follow-up. The long-term success rates of the 2 groups were 87.0% and 61.1% (*p* < 0.05), respectively.

SND occurred in 2 patients with PCS and 4 patients with SS (4.3% vs. 22.2%, *p* < 0.05). Among them, 1 patient in the PCS group and 3 in SS group had SND occurrence during or shortly after the procedure. The one in PCS group had AFL and SMAT, and ablation was performed at CTI and from the lateral scar to TA. In the 3 patients in the SS group, 1 had 5 kinds of AT, and extensive ablation was performed at CTI and from posterior scar to SVC and IVC; 1 had 3 kinds of AT and had scars in posterior and lateral walls, in whom atrial electrical standstill occurred after ablation from posterior scar to IVC, lateral scar to TA and TA to IVC. Another patient had a broad scar in the lateral and anterior walls, and AT was confirmed as focal mechanism with origin from the superior margin of the scars, in whom junctional rhythm appeared during tentative ablation at the earliest activation. A large area of scar and extensive ablation might be possible reasons for SND. All of the patients with SND underwent ICD implantation except for 1 patient, whose heart rate maintained at 30–50 bpm with no significant symptoms.

## 4. Discussion

After retrospectively analyzing and comparing the characteristics of ATs in patients with PCS and SS, there were several findings in our research: (1) Patients with SS have more kinds of ATs than those with PCS. (2) Typical AFL is prevalent in both groups, whereas the SMAT and FAT are more common in patients with SS. (3) Despite of more linear ablation, patients with SS have higher recurrences and lower long-term success rates. (4) Patients with SS had more SND after ablation.

The findings in this study are consistent with previous reports that the most common atrial tachycardia in patients with PCS was AFL, whereas SMAT was seen in less than half [2,8,9,12]. It has been demonstrated that slow conduction area for AFL was located in CTI in patients with normal atrial substrate, but was in the lateral wall of TA in those with PCS [13]. In addition, the longitudinal diameter of the right atrium was remarkably longer in the PCS group. The retarded conduction in incisional scar and its vicinity and the elongated conduction pathway may facilitate the atrial myocytes in front of the activation wavefront to recover from the refractory period and the reentrant activation can persist, which provides the electrophysiologic element for reentry [6,12,13]. Furthermore, the right atrial incision, usually from the posterior part of RAA towards IVC, is parallel to tricuspid annulus, so it can serve as an acquired obstacle to form a conduction pathway. This facilitates the atrial electrical propagation along a specific route and provides the anatomical basis for reentry. The two elements mentioned above can explained the high incidence of AFL. In addition, the slow conduction and central obstacle caused by incisional scar are also advantageous to the occurrence of SMAT [7]. However, SMAT here was only seen in 39.1% patients with PCS, significantly lower than AFL. Some research found that the occurrence of SMAT in patients with corrected CHD was associated with the complexity of CHD and the length of right atrial incision; the more complex the anatomy and the longer incision are, the higher the occurrence of SMAT [14]. In 35 patients that had undergone surgery for CHD in our study, 57.1% had moderate complexity of CHD whereas only 8.5% had severe complexity according to the ACHD guideline, which may explain, to some extent, the relatively low occurrence of SMAT. Yang et al. found an incision longer than 51.5 mm was the independent predictive factor for incisional reentrant tachycardia [15]. This can be explained by a longer reentrant circuit around the incision, which make the atrial myocyte in the circuit free from a refractory period and maintains stable reentry. However, recently, a study from three European centers found the extent of surgical atrial repair and complexity of CHD exerted no influence on ablation outcomes [9].

In patients with SS, both SMAT and AFL show a high occurrence, especially the SMAT [10]. This could be related to the characteristics of SS. Different from incisional scars locating mainly at the lateral wall, SS was more diffused and scattered [3,4,5]. It was the following elements that constituted the requirements for reentrant tachycardia and made a significantly higher occurrence and more variety of SMAT: the isthmus and slow conduction area between two scars or between the scar and anatomical barriers, the central obstacle formed by various scars and the low-voltage area with time-dependent decremental property [16]. This can also explain the high occurrence of AFL. Furthermore, the occurrence of FAT was also higher. Previous studies found FAT in patients with SS can be initiated or terminated by atrial pacing, indicating micro-reentrant mechanisms [4,5,17]. We considered that the low-voltage areas on the edge of small and heterogenetic scars can form slow conduction zones and a reentrant isthmus, which can cause small reentry and micro reentry and result in the higher occurrence of FAT in patients with SS.

Some novel ablation methods, such as isolation of the right atrial free wall and atrioventricular node modulation, were proven to be feasible in AT treatment, but these methods did not target the critical isthmus in the substrate of ATs [18,19]. In this research, all radiofrequency ablation was tailored according to specific AT mechanisms and substrates. The common ablation position in patients with SS was CTI and scar to IVC, whereas other linear ablations were also performed, such as from scar to SVC or TA, and scar fusion. With more linear ablation, the long-term success rate of patients with SS in our research was 61.1%, significantly lower than in patients with PCS [20]. Expansion of the SS area is often found in patients with recurrence, and the septal scar can even expand from the right atrium to left atrium [5]. Some researches proposed tachycardia-mediated atrial structure remodeling as a potential mechanism for atrial fibrosis, whereas others considered that SS expansion may be caused by the progression of potential primary diseases [21,22]. A study performed on canine hearts found that sustained AFL can shorten the atrial refractory period and the action potential duration, but will not cause atrial fibrosis or activation of inflammation and extra-cellular matrix [22]. It indicated right atrial scar formation was more likely caused by the atrial cardiomyopathy which causes AFL, rather than AFL itself. Myocardial dysplasia, congenital cardiomyopathy and some acquired cardiomyopathies caused by post-inflammation scar, amyloid deposition and immunocomplex deposition are the possible reasons for atrial scar formation [23]. In some diseases such as systemic lupus erythematosus, inflammatory cell infiltration can develop into necrosis of cardiomyocytes and fibrose replacement, finally causing myocardial scars [15,24]. With the progression of primary disease, the ever-expanding myocardial scars may produce new reentrant circuits and cause arrhythmia recurrence. However, there is controversy over the underlying mechanisms of scar expansion, and further research is needed.

SND was found both in patients with PCS and patients with SS in our research, but those with SS had a significantly higher occurrence [17,25,26]. SND is the pathological change in the sinoatrial node and its adjacent tissue, causing disorders of sinoatrial pacing and conduction functions [27]. The common reasons for SND in patients with PCS or SS are as follow. First, the atriotomy incision of cardiac surgery may injure the sinus node or its blood supply [28]. Second, multiple linear ablations combined with the surgical scars or the SS can cause atrial compartmentation, enclosing the sinus activation in a limited area and preventing its further propagation, which displayed as partial atrial standstill [7]. Third, extensive right atrium lesions in patients with SS might trespass the sinus node or influence the atrial electrical activation, leading to SND, as revealed by previous studies that the amplitude of P wave in lead II and the mean voltage of right atrium were significantly lower in SND patients, indicating a diseased right atrium [3,4].

### Limitations

There are several limitations in this research. First, this is a retrospective study in a single center and the time span is long, from 2013 to 2019, so the cohort is heterogenous and the electrophysiological protocol were not completely uniform in all cases. In earlier times, electroanatomical mapping was performed using an ablation catheter and the number of mapping points of the right atrium was relatively small, even less than 300 in some cases, so the electroanatomical models of the right atrium were coarse and less precise. It was not until later that some high-density mapping tools such as PentaRay multipolar mapping catheter and Rhythmia mapping system had become available that more precise and reliable electroanatomical mapping was able to be performed. Because of the distinct devices and techniques used in different periods, the area and proportion of scars in the two groups were not analyzed; instead, the location of the scars and the characteristics of related tachycardias were described. Second, the sample size was small, so we did not perform the comparison between different mapping systems or within the same mapping system. Third, although transthoracic echocardiograms were performed in all patients with SS and no significant structural abnormalities were found, further examinations were not performed, such as cardiac magnetic resonance images and genetic testing, so the underlying diseases for SS in our research remain unknown. Fourth, only patients with right atrial tachycardia were included in this research, and those with left atrial tachycardia were excluded. Thus, the influence of PCS and SS on the left atrium and on other arrhythmias remains unknown.

## 5. Conclusions

AFL is prevalent in both patients with PCS and patients with SS, and routine CTI ablation was recommended in these populations. Compared with patients with PCS, patients with SS have more ATs, especially with higher occurrence of SMAT and FAT, and more linear ablation is needed. Patients with SS have lower long-term success rates and higher incidences of SND.

## Figures and Tables

**Table 1:** Categories of initial structural heart disease in patients with cardiac surgery.

Heart Diseases	Number (*N* = 46)
Congenital heart diseases	35
Trilogy of Fallot	1
Tetralogy of Fallot	8
With atrial septal defect	1
Double outlet of right ventricle	3
With patent ductus arteriosus	1
Corrected transposition of great arteries	2
With persistent left superior vena cava	1
With dextrocardia	1
Ebstein’s anomaly	2
With atrial septal defect	1
Anomalous pulmonary venous connection	2
With atrial septal defect	2
With cor triatriatum	1
Pulmonary artery stenosis	3
With atrial septal defect	1
Atrioventricular septal defect	1
Ventricular septal defect with patent ductus arteriosus	1
Simple ventricular septal defect	5
Simple atrial septal defect	7
Valvular heart diseases	7
Mitral valve disease	6
Tricuspid valve disease	1
Cardiac tumors	4
Left atrial myxoma	4

**Table 2:** Baseline characteristics of two groups.

	Prior Cardiac Surgery (N = 46)	Spontaneous Scar (N = 18)	*p* Value
Male	22 (47.83%)	9 (50.00%)	0.88
Age	46.00 (34.00, 52.25)	56.50 (54.00, 67.25)	0.00 *
Sustained atrial tachycardia	25 (54.3%)	6 (33.3%)	0.13
Prior ablation	6 (13.0%)	4 (22.2%)	0.45
Atrial tachycardia	5 (10.9%)	2 (11.1%) **	1.00
CTI ablation	5 (10.9%)	2 (11.1%) **	1.00
Others	1 (2.2%)	3 (11.1%) **	0.06
SVT	0 (0.0%)	2 (11.1%)	0.08
VT	1 (2.2%)	1 (5.6%) **	1.00
Other prior history			
Hypertension	5 (10.9%)	2 (11.1%)	1.00
Diabetes Mellitus	2 (4.3%)	1 (5.6%)	1.00
Coronary Heart Disease	1 (2.2%)	1 (5.6%)	0.49
Echocardiography			
Left atrium (mm)	38.04 ± 7.20	35.72 ± 4.04	0.17
Right atrium (mm)			
Horizontal diameter	48.00 (38.00, 57.00)	47.00 (40.50, 55.50)	0.85
Longitudinal diameter	53.00 (33.00, 66.00)	39.00 (34.00, 46.50)	0.02
Left ventricle (mm)	45.93 ± 7.22	46.89 ± 3.88	0.61
Right ventricle (mm)	20.00 (16.00, 25.00)	17.00 (14.50, 17.50)	<0.01
LVEF	0.58 ± 0.09	0.61 ± 0.06	0.17

CTI: Cavo-tricuspid isthmus. * Data was compared by Mann–Whitney U test. ** One patient had both prior ablation for AFL and VT.

**Table 3:** Outcomes of long-term follow-up.

	Prior Cardiac Surgery (N = 46)	Spontaneous Scar (N = 18)	*p* Value
Follow-up duration	63.0 (49.8, 81.0)	70.0 (48.5, 94.0)	0.25
Sinus node dysfunction	2 (4.3%)	4 (22.2%)	<0.05
ICD implantation	3 (6.5%)	4 (22.2%)	0.17
Recurrence	7 (15.2%)	8 (44.4%)	0.03
Redo ablation	1 (2.2%)	1 (5.6%)	0.49
Long-term success	40 (87.0%)	11 (61.1%)	<0.05

## Data Availability

The data presented in this study are available on request from the corresponding author.
